# Excision of Pyogenic Granuloma (PG) With Diode Laser on the Lower Lip in an Unusual Location: A Case Report

**DOI:** 10.7759/cureus.44319

**Published:** 2023-08-29

**Authors:** Luciane Hiramatsu Azevedo, Luana Maria Ferreira Nunes, Letícia Drumond de Abreu Guimarães, Pedro Cardoso Soares

**Affiliations:** 1 Faculty of Dentistry, University of São Paulo, São Paulo, BRA; 2 Hospital Dentistry, Hospital Albert Einstein, São Paulo, BRA; 3 Oral Pathology, University of São Paulo, São Paulo, BRA

**Keywords:** aesthetic zone, lower lip, oral biopsy, photobiomodulation, pyogenic granuloma, 940 nm diode laser, laser procedure

## Abstract

Pyogenic granuloma (PG) is a tumor with regular growth expressed usually in the oral cavity, expressing characteristics of a non-neoplastic lesion. The first treatment option is surgical excision, which can be proceeded with surgical diode lasers (940 nm). This case report focuses on the surgical excision of a PG located in the lower lip using diode lasers. Post-operative follow-up of 6 months demonstrated adequate healing without esthetical compromise and no lesion recurrence, showing that diode lasers can be a safe and effective alternative for PG removal.

## Introduction

Pyogenic granuloma (PG) is an acquired reactive benign vascular proliferation that develops as a small erythematous papule in the skin or oral mucosa surface. It is frequently caused by low-level persistent infection, small trauma, poor oral hygiene, or even hormonal disturbance. It shows a higher occurrence in gingiva [1]. It can be also expressed in lips, tongue, soft and hard palate, and also oral mucosa [2-3]. This lesion usually presents asymptomatic and slow-growing characteristics, despite the fact some cases can present a faster growth, while others remain stable for a long time. The lesion size varies between a few millimeters and centimeters, rarely becoming larger than 2 cm. It can be seen at any given age, usually observed more frequently between 10 and 40 years of age, with peak expression during the second decade of life. It is usually more expressed in young females, possibly due to the hormonal effect in vascularization caused by estrogen and progesterone [4].

As it is not classified as a neoplastic lesion, excisional therapy is indicated. The patients must be aware of the risk of recurrence, which is 14.8% using traditional excision methods in a one-year period. Such events are probably linked with a lack of complete lesion removal or/and failure in controlling etiological factors exposure responsible for lesion recurrence [5-6].

In addition to traditional surgical techniques, lasers are described as alternative tools for PG surgical removal. There are different types of lasers that can be used for surgical removal of PG (Diode Lasers, Nd:YAG, Er:YAG, CO2, and also flashlamp pulsed dye laser). Sclerotherapy is also another viable option for PG management [7-11]. 

In this case report the authors opted for diode laser usage in the 940 nm wavelength. It presents a great alternative for PG treatment due to the characteristics obtained when using such a device, additionally becoming an optimal tool for PG removal in esthetic sensible areas, such as gingival esthetical zones and vermilion region of the lips [1, 12-13].

## Case presentation

**Figure 1:**
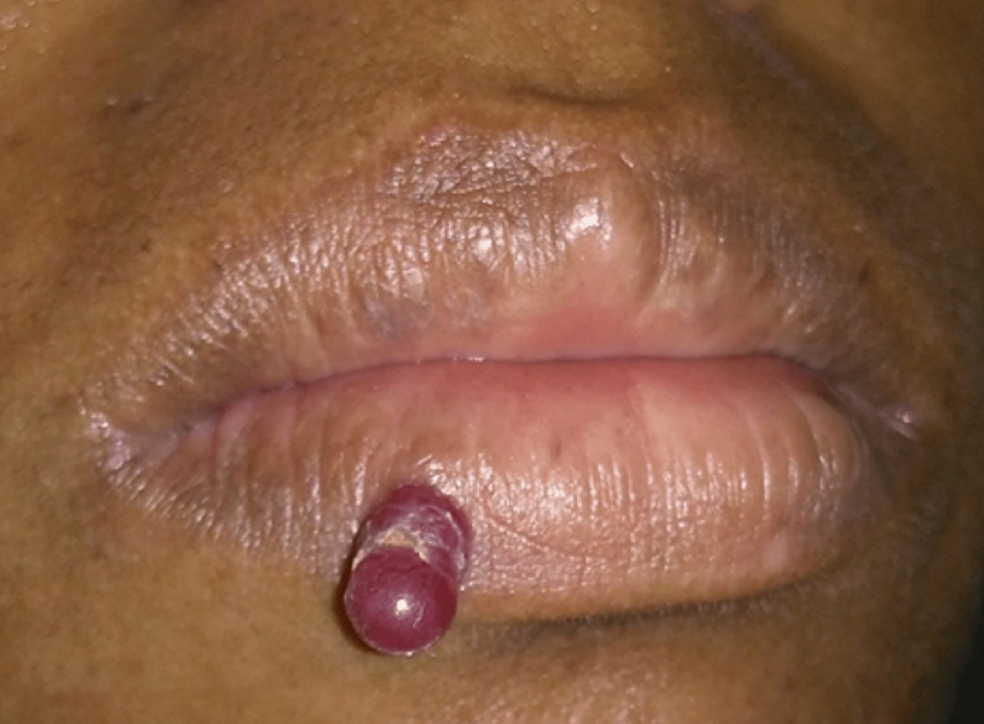
A well-defined reddish blue, firm in consistency, non-compressible lesion measuring 2 cm x 1 cm was observed on the right lower border of the lips.

A 37-year-old woman presented to the Special Laboratory of Lasers in Dentistry at the School of Dentistry of the University of São Paulo - City of São Paulo, State of São Paulo, Brazil - with chief complaint of edema on the right lower lip border. She mentioned that the lesion first appeared 4 months ago, with a gradual increase in size since then. The patient did not report any trauma in the specific region that could lead to such lesion development. Clinical examination revealed an erythematous sessile nodule of 2 cm x 1 cm located in the vermillion region of the inferior lower lip (Figure 1). 

The patient did not report any previous medical condition (diabetes, cardiocirculatory, hormonal alterations, smoking history) or continuous medication use. Pregnancy was also excluded with appropriate blood tests. All other blood test parameters evaluated were considered normal. Concerning dental history, the patient presented overall good dental/oral condition. 

The lesion had a firm consistency to touch, it was not compressible, and had no signs of blood pulsation. The patient did not report spontaneous bleeding, pain or exudate, despite bleeding when the lesion was traumatized. 

The two diagnostic hypotheses were: oral varicose vein or pyogenic granuloma, based on the clinical aspect and color of the lesion. 

An excisional biopsy was performed, based on the diagnostic hypothesis. Written informed consent was obtained prior to the excision of the lesion.

**Figure 2:**
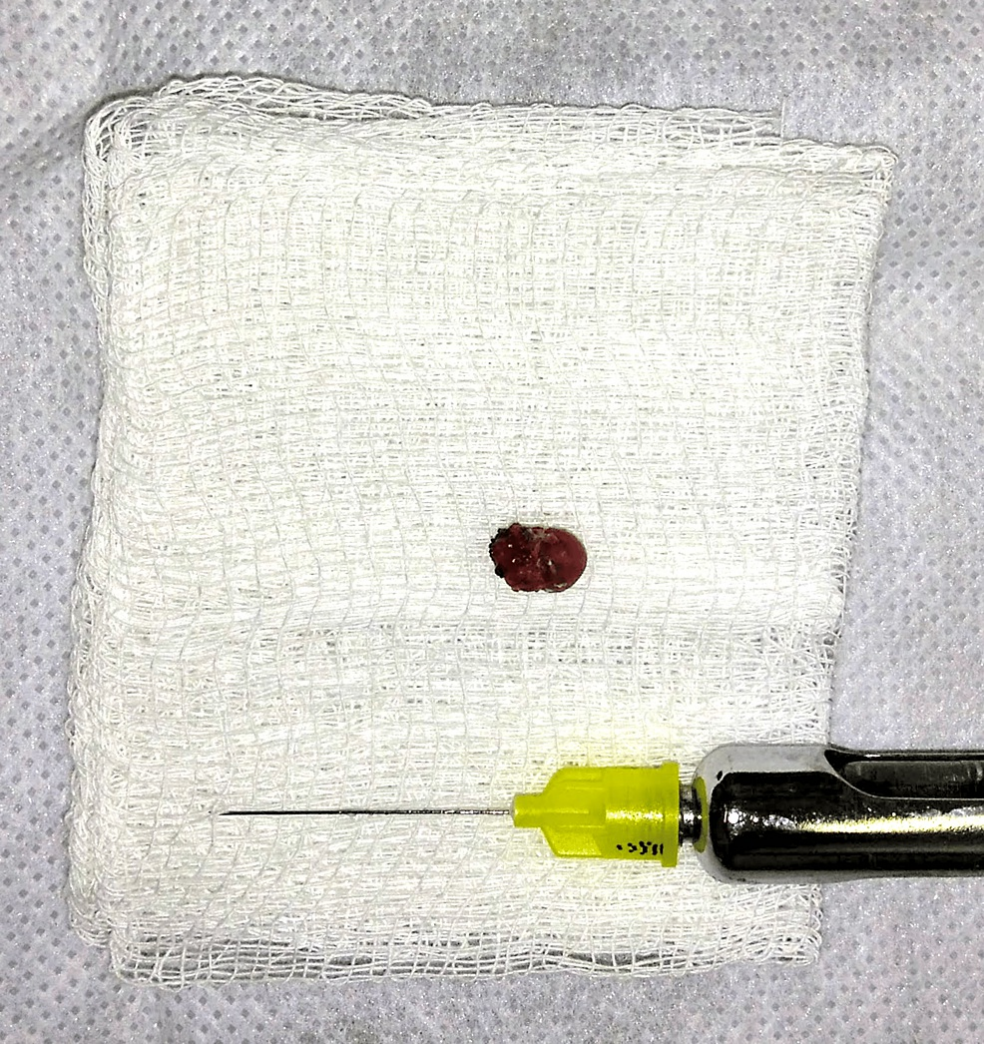
Clinical aspect of the lesion after excisional biopsy using diode lasers (980 nm, 1.5 W - CW).

A diode laser with 940 nm wavelength (EpicX ™, Biolase, USA) was used for tissue removal, under local anesthesia (Lidocaine 2% w/epinephrine 1:100.000 I.U). Irradiation was performed with a handpiece connected to a disposable fibertip (Tip E3-4 - 300 micrometers - EpicX ™, Biolase, Foothill Ranch, CA), using continuous wave with a 1.5 W power. The fibertip was activated with blue occlusal paper. The incision with diode laser was performed in the contact region between the lesion and lower lip tissue, performing complete lesion removal (Figure 2).

**Figure 3:**
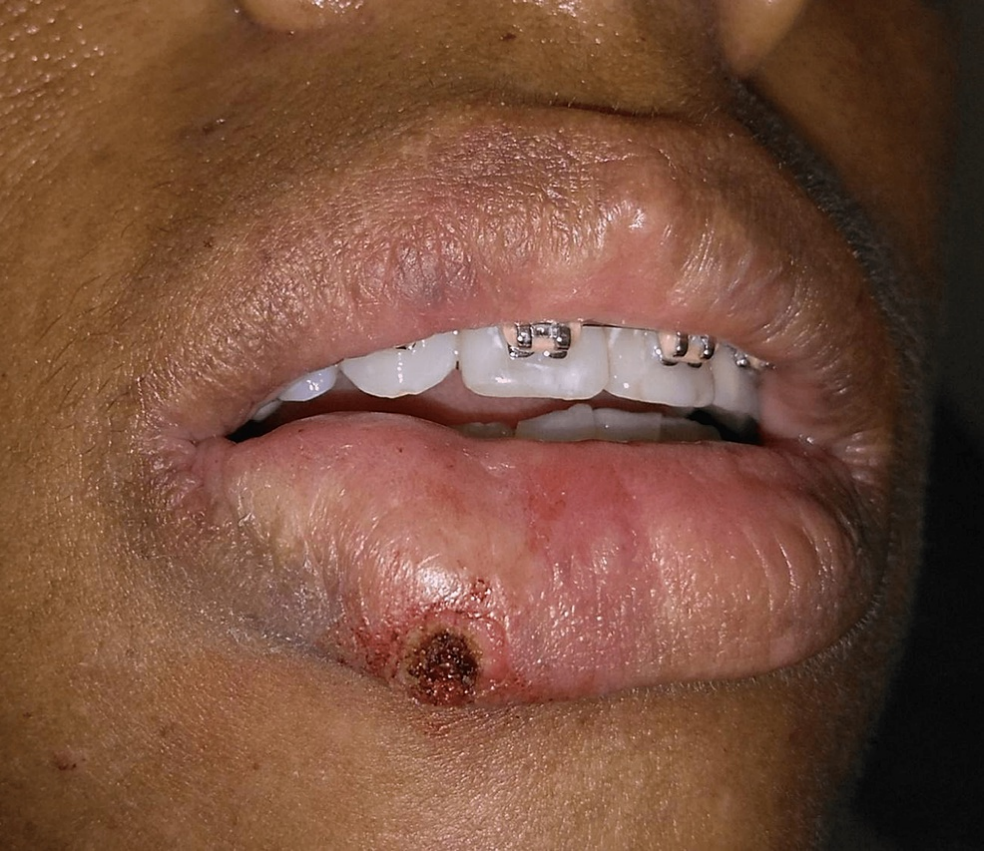
Diode laser showed adequate combination of homeostasis in parameters used for PG removal, helping the surgeon visualize the operative field and promoting a fast and safe procedure. PG, pyogenic granuloma

Diode laser surgery promoted an ideal homeostasis facilitating the surgical procedure (Figure 3). Analgesic (Acetaminophen 750 mg - 6/6 h) was prescribed in case of pain, if necessary.

**Figure 4:**
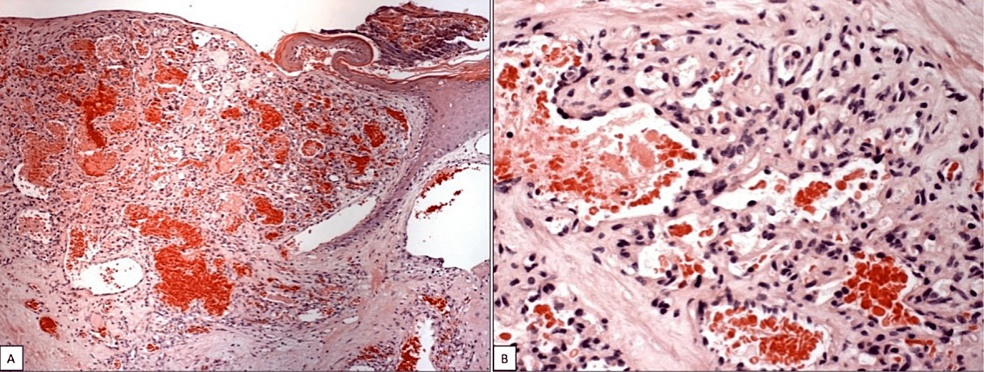
(A) Ulcerous area exhibiting diffuse endothelial cells proliferation that opens vascular spaces, 100x magnification (Hematoxylin and Eosin). (B) Endothelial cells with lobular pattern that opens vascular spaces, sometimes obliterated, 400x magnification (Hematoxylin and Eosin).

The histopathological analysis after the surgery showed an ulcerous surface in a stratified squamous epithelium, along with parts of the lesion demonstrating a hyperkeratinized stratified squamous epithelium and other areas demonstrating atrophic squamous epithelium, covering partially the surrounding connective tissue. Additionally, neovascularization was evident (Figure 4A). 

These blood vessels sometimes were organized in lobular format (Figure 4B). An infiltrate of inflammatory cells, mainly with neutrophils, plasmocytes, and lymphocytes is evident. All of these characteristics confirmed the PG diagnosis.

**Figure 5:**
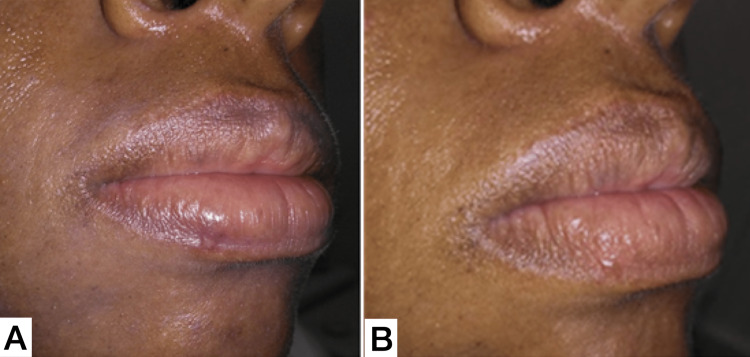
(A) 1 month follow-up after PG removal with diode laser. (B) 6 months follow-up after PG removal with diode laser, without signals of lesion recurrence. PG, pyogenic granuloma

Complete healing was observed after 4 weeks (Figure 5A), without complications. Discomfort and formation of cicatricial scar tissue were minimal after the surgical procedure. After 32 weeks, the patient returned and there was no sign of recurrence (Figure 5B).

## Discussion

Pyogenic granuloma (PG) is a neoplastic benign vascular malformation that results in soft tissue excessive hyperplasia. The prevalence of this condition is higher between the second and fifth decade of life, presenting higher expression in women. Despite the lack of a precise etiopathological mechanism, local trauma or chronic irritation may lead to excessive tissue repair stimuli leading to condition development. Clinically, the lesion can present itself as nodules or papulae, ranging from a few millimeters to a few centimeters [5]. PG has a broad spectrum of lesions for differential diagnosis, such as hemangioma, peripheral ossifying fibroma, Kaposi sarcoma, lymphangioma, peripheral giant cells granuloma, and angiosarcoma. The diagnosis confirmation is made only by histopathological evaluation [3]. In this specific case report, the cause for the development of the lesion was not clear, and consequently, it was not possible to diagnose, manage, or remove the factor that developed the lesion. 

Different therapeutic modalities can be utilized for PG treatment, according to the size and location of the lesion. In this case, the authors opted for diode laser surgery (980 nm) removal due to the advantages presented by this technique. There are a great variety of advantages of diode lasers in the near infrared spectrum (800-1000 nm) usage when compared to the traditional excision technique, which is: better visualization of the surgical field caused by the hemostatic effect of the diode laser wavelength (800-1000 nm) caused by the absorption of energy by pigments such as hemoglobin; reduced percentage of lesion recurrence by the elimination of viable cells located in the lesion transitional area to the sound tissue; photobiomodulation of the surrounding tissues reducing post operative pain and discomfort; reduced risk of esthetical compromise and scar formation in esthetical sensible areas; reduction in the number of the bacteria at the intervention site; and also reduced risk of postoperative complications. Furthermore, other possible options for PG treatment are sclerotherapy, cauterization with electrocautery, and removal using other types of laser wavelength [3, 10]. The type of laser wavelength plays a significant role clinically, once each wavelength promotes different interactions with target tissues, consequently showing different results during surgery. Homeostasis, ablation, and other photothermal effects and posterior photobiomodulation in the surrounding areas of intervention are some of the effects that are different between different devices available. Another aspect to be considered is the cost, as diode lasers have the lowest acquisition price when compared to other types of lasers (Nd:YAG, Argon, Er:YAG, CO2) [9, 11-12]. 

Energy delivery to the target tissue is arguably the most important factor that must be observed. In this case report, the laser fiber was activated with blue occlusal paper and the power output was 1.5 W. These parameters promoted adequate cutting efficiency and demonstrated accordance with the benefits described in the literature. These aspects must be carefully considered, once even with the proper laser, if the parameters are not adequate, major damage to surrounding tissues and scar formation inevitably will occur. 

Finally, the surgeon must be aware of all variables when using diode lasers to obtain the best results when working with diode lasers, aware of the risks involved, and the potential benefits to the patient.

## Conclusions

Diode lasers are a safe and effective option for PG management in esthetic zones, showing no scar formation and low discomfort for the patients. The relatively low cost of diode laser operation along with portability, are the additional advantages of this technique.
